# Exploring the Dimensions of Movement-Specific Reinvestment From Personal Characteristics Perspectives

**DOI:** 10.3389/fpsyg.2021.716945

**Published:** 2021-09-24

**Authors:** Masato Kawabata, Kuniyasu Imanaka

**Affiliations:** ^1^Physical Education and Sports Science Academic Group, National Institution of Education, Nanyang Technological University, Singapore, Singapore; ^2^School of Human Movement and Nutrition Sciences, The University of Queensland, Brisbane, QLD, Australia; ^3^Department of Health Promotion Sciences, Graduate School of Human Health Sciences, Tokyo Metropolitan University, Hachioji, Tokyo, Japan

**Keywords:** reinvestment, confirmatory factor analysis—CFA, exploratory structural equation modeling—ESEM, mediation analysis, latent class factor analysis (LCFA), trait

## Abstract

The purposes of the present study were three-fold: to examine (a) if the movement-specific reinvestment responses should be represented as two dimensional constructs, (b) whether dichotomization of the movement-specific reinvestment responses are appropriate, and (c) how the two dimensions are associated with relevant psychological concepts. To conduct a comparative examination of the MSRS structure in two Asian samples, participants were 236 Japanese university students (136 men, 100 women; *M*_age_ = 18.0, *SD* = 1.6) and 328 Singaporeans (167 men, 161 women; *M*_age_ = 21.8, *SD* = 1.8). After examining the factor structure of the movement-specific reinvestment responses for the first purpose, latent class factor analysis was conducted for both samples for the second purpose. For the third purpose, correlation analysis and mediation analysis were conducted for a part of the Singaporean sample. Through a series of latent class factor analysis, four and three classes were identified for the Japanese and Singaporean samples, respectively. For both samples, the patterns of the item-average scores for the two movement-specific reinvestment dimensions were parallel among the classes. Conscious Motor Processing was positively associated with mental toughness, intrinsic regulation, integrated regulation, mastery-approach and task goal orientations, and dispositional flow, whereas Movement Self-Consciousness was positively related with stress and mastery-avoidance goal orientation. The findings of the study supported (a) the two-dimensional representation of the movement-specific reinvestment responses, but did not fully support (b) the practice of dichotomization of the movement-specific reinvestment responses, and indicated that (c) at the trait level, Conscious Motor Processing and Movement Self-Consciousness were associated with positive and negative psychological constructs, respectively.

## Introduction

There are many occasions in which individuals are required to carry about complex tasks well under stressful situations and playing sport under pressure is one of them. Some individuals perform poorly under pressure whereas others show superb performance by handing pressure. It is crucial to understand the underlying mechanisms of movement disruption under pressure so that everybody, including professionals such as surgeons and pilots, is able to perform well under stressful situations. In the theory of reinvestment, Masters and colleagues (Masters, 1992; Masters et al., 1993) suggested that the performer's attempts to consciously monitor and control the mechanics of movements (i.e., *reinvestment*) could disrupt relatively automated motor processes if conscious monitoring and control mechanisms are employed improperly. They argue the involvement of conscious monitoring and control mechanisms in motor processes depends on situational contexts, such as psychological pressure or individual personality differences (Masters and Maxwell, 2008; Malhotra et al., 2015).

The Movement-Specific Reinvestment Scale (MSRS: Masters et al., 2005) is a 10-item self-report instrument to quantify individual's personality tendency for reinvestment in movement-specific situations. The MSRS consists of two dimensions: Movement Self-Consciousness (MS-C) and Conscious Motor Processing (CMP). MS-C is characterized by “concern about *style* of movement and about making a good impression when moving in public” (e.g., “I am concerned about my style of moving”). CMP is characterized by “contemplation of the process of movement” (e.g., “I am aware of the way my body works when I am carrying out a movement”) and consistent with reinvestment theory (Masters and Maxwell, 2008).

The structure of the MSRS has been examined for different language samples using the original English or translated versions (French, German, and Chinese). The two-factor structure was generally supported within the confirmatory factor analysis (CFA). Masters et al. (2005) reported that a moderate relationship was observed between CMP and MS-C (British Royal Air Force sample: *r* = 0.53; Student sample: *r* = 0.43) and considered the two factors were independent. However, the association between CMP and MS-C was reported based on Pearson's correlation and the latent correlation in the previous MSRS studies (Masters et al., 2005; Laborde et al., 2014, 2015; Ling et al., 2016). Pearson's correlations based on scale scores tend to be lower than CFA-based latent correlations that are more sensitive to measurement error (see Mallett et al., 2007, for further details of this tendency). Consequently, it was unclear whether CMP and MS-C were distinguishable for their non-English speaking samples within the CFA framework.

Duda and Hayashi (1998) cautioned that if research on the psychological dimensions of sport and exercise behavior is delimited only to the mainstream group (i.e., white, college-aged, middle-class, and mostly male individuals), such studies are opposed to the very essence of scientific inquiry. They also argued cross-cultural studies make important contributions to the development of theory and knowledge in the field. Although the MSRS has been widely used in the literature, the examination of the MSRS structure for non-Western samples (e.g., Asian) has been limited to its Chinese version (Ling et al., 2016). Thus, it is desirable to examine the MSRS structure for non-Western samples. To this end, it would be more thorough to conduct a comparative investigation into the MSRS structure between two Asian samples by using the original English and translated versions of the MSRS (e.g., English and Japanese versions; see Kawabata et al., 2017).

In the reinvestment research, the entire group was often divided into two subgroups (high vs. low reinvestment groups) by conducting a mean or median split (e.g., Laborde et al., 2015). However, the approach is practically problematic as dichotomization is specific to the mean or median of each sample. With this approach, it would be possible that an individual with the MSRS score of X is categorized as a low-investor in a sample but classified as a high-investor in another sample. The practice of dichotomization based on the midpoint split approach is also considered statistically problematic because the categorization of any continuous variable leads to the reduction of the data precision and substantial loss of statistical power of analyses (MacCallum et al., 2002). MacCallum and colleagues strongly argue that even if cases in which dichotomization of quantitative scales and analysis of group differences are truly appropriate, the practice of dichotomization should be supported by compelling results from taxometric analyses (person-centered approaches). To authors' knowledge, taxometric analysis was never conducted on the MSRS responses.

Masters and Maxwell (2008) considered that reinvestment could be prevented or reduced through emotion control training (Roger and Masters, 1997), distraction techniques (e.g., Wulf et al., 2007), or acclimatization (Beilock and Carr, 2001). Given that reinvestment may be a characteristic of personality (Masters et al., 1993), it is important to comprehensively understand what factors are associated with developing personal characteristic of reinvestment. For example, Laborde et al. (2015) found that parental criticism appears to be related to developing a tendency to reinvest regarding cognitive decision. Furthermore, they found that the CMP score was positively associated with motor imagery ability and argued that reinvestment should not be always considered to be negative due to its link to choking under pressure, but it could be beneficial, depending on the task being performed. It is well-known that motivational climates produced by coaches, peers, and parents strongly influence athlete's emotions, motivational style, goal orientations, and behaviors in sport settings (e.g., Duda et al., 2014; Ntoumanis and Mallett, 2014). Exploring what social and psychological factors are associated with developing personal tendency for reinvestment would be beneficial to understand reinvestment better and prevent it under stressful situations. However, research on the association between movement-specific reinvestment and other psychological constructs is scarce except for the studies by Laborde et al. (2015) and Ling et al. (2016). Therefore, it was considered important to address the research gaps.

The purposes of the present study were three-fold: to examine (a) if the movement-specific reinvestment responses should be represented as two dimensional constructs, (b) whether dichotomization of the movement-specific reinvestment responses are appropriate, and (c) how the two dimensions are associated differently with relevant psychological concepts, such as emotions, motivational regulations, goal orientations, and positive psychological experiences. To minimize the negative outcomes of reinvestment and promote its potential benefits, it was necessary to understand the relationships between the MSRS dimensions and the relevant psychological constructs comprehensively.

## Methods

### Participants and Procedures

To conduct a comparative examination of the MSRS structure in two Asian samples, participants in this study were 236 Japanese university students (136 men, 100 women; *M*_age_ = 18.0, *SD* = 1.6, the range of age: 18–27 years old) and 328 Singaporeans (167 men, 161 women; *M*_age_ = 21.8, *SD* = 1.8, the range of age: 19–32 years old). They were recruited from a health science course at a Japanese university or from psychological courses at a Singapore university. The lecturer of the course administered a survey set during a lesson. Participants were asked to complete the survey set by following instructions on how to respond to each scale, which were written at the top of each scale. Ethical approval was obtained from an Institutional Review Board of Nanyang Technological University, Singapore [IRB-2013-11-004] before the data collection. Participation was voluntary and informed consent was received from each participant. All participants completed the demographic sheet and the self-report measure of reinvestment. However, other psychological measures were only completed by a part of Singaporean sample (*n* = 53) who enrolled in an advanced psychological course and participated in a competitive sport.

### Measures

The following measures were employed to explore the associations between movement-specific reinvestment and other psychological constructs such as emotions/subjective feelings (anxiety, stress, vitality, confidence), motivational style, goal orientations, and positive experiences (flow) in sport settings.

#### Reinvestment

The Movement Specific Reinvestment Scale (MSRS: Masters et al., 2005) is a 10-item self-report instrument to measure the personal tendency to consciously attend to and control movements, which consists of two factors: CMP and MS-C (see the Introduction for the details of the MSRS). Participants were asked to indicate the degree to which they generally agreed with the statement of each item on a 6-point Likert-type scale, ranging from 1 (*strongly disagree*) to 6 (*strongly agree*).

The English version of the MSRS was administered to Singaporeans. For the Japanese, the MSRS was translated into Japanese by following the team approach used by Kawabata et al. (2008). The team approach was proved to be valid and efficient in Kawabata et al. (2008) and Kawabata et al. (2017). The first author who is a native Japanese speaker and fluent in English translated the scales into Japanese. All items were most carefully translated or adapted to minimize cross-cultural/cross-linguistic bias. Subsequently, the second author who is also a native Japanese speaker and a faculty member in sport psychology and his graduate students confirmed that all the items were appropriately translated into Japanese.

#### Anxiety and Stress

The anxiety and stress subscales of the Depression Anxiety Stress Scales-21 items (DASS-21: Lovibond and Lovibond, 1995) were used to measure participant's perceived anxiety and stress levels. Participants were asked to indicate the degree to which each statement applied to them over the last week on a 4-point Likert scale ranging from 1 (*did not apply to me at all*) to 4 (*applied to me very much or most of the time*). The DASS-21 is not a trait measure but a state measure. However, it was used in Kawabata et al.'s (Kawabata et al., 2020) study to examine the associations between anxiety/stress and a personality trait of mental toughness.

#### Subjective Vitality

The Subjective Vitality Scale (SVS: Ryan and Frederick, 1997) is a seven-item self-report instrument that is designed to assess feelings of energy and vitality. There are two versions (i.e., state and trait levels). According to Kawabata et al.' (Kawabata et al., 2017) study, the five-item trait-level model of the SVS was used in the present study. Participants were asked to indicate the degree to which the statement of each item was true for them “in general in their life” on a 7-point Likert-type scale ranging from 1 (*not at all true*) to 7 (*very true*).

#### Mental Toughness

The Mental Toughness Questionnaire-48 (MTQ: Clough et al., 2002) is a self-report instrument designed to measure one's level of mental toughness. The 6-item Very Short MTQ (Kawabata et al., 2020) is a refined and abbreviated version of the MTQ to measure the six aspects of mental toughness proposed by Clough et al. Participants were asked to indicate the degree to which they generally agreed with the statement of each item on a 5-point Likert scale, ranging from 1 (*strongly disagree*) to 5 (*strongly agree*).

#### Dispositional Flow

The short Dispositional Flow Scale-2 (S-DFS-2: Jackson et al., 2008) is the nine-item trait-level measure to assess the frequency of flow experience during participation in an activity. Flow is a metaphorical term to illustrate the feeling that people similarly when they are acting with focused and intense involvement (Kawabata and Mallett, 2011). Participants were asked to indicate how often they generally experience the characteristics of flow in their sport on a 5-point Likert scale, ranging from 1 (*never*) to 5 (*always*).

#### Sport Motivation

The Sport Motivation Scale-II (SMS-II: Pelletier et al., 2013) is the 18-item trait-level measure, consisting of six subscales (three items each), that corresponds to the six forms of motivation proposed in the self-determination theory framework. Participants were asked to indicate the degree to which their own personal reasons for participating their sport corresponded the listed reasons on a 7-point Likert-type scale, ranging from 1 (*not at all true*) to 7 (*very true*).

#### Goal Orientations

Two measures were used to assess participant's goal orientations. The Task and Ego Orientation in Sport Questionnaire (TEOSQ: Duda and Nicholls, 1992) is a 13-item instrument to measure task and ego goal orientation toward sport. Participants were asked to indicate the degree to which they agree with statements about their success in sport on a 7-point Likert-type scale, ranging from 1 (*strongly disagree*) to 7 (*strongly agree*).

The Achievement Goal Questionnaire-Revised (AGQ-R: Elliot and Murayama, 2008) is a 12-item trait-level measure to assess achievement goals as proposed in the 2 × 2 goal achievement framework. Participants were asked to indicate the degree to which their own reasons for attending university classes corresponded the listed reasons on a 5-point Likert scale, ranging from 1 (*strongly disagree*) to 5 (*strongly agree*).

### Data Analysis

To examine the factor structure of the MSRS responses for the first purpose, confirmatory factor analysis (CFA) and exploratory structural equation modeling (ESEM) were conducted with *M*plus (Version 8.2; Muthén and Muthén, 1998–2019) based on Mplus robust maximum likelihood estimation (MLR). Missing data (< 0.5% missing responses for each scale) were treated by the Expectation Maximization Algorithm (Bentler, 2006). In the CFA model, each item was allowed to load on only one target factor and all non-target cross-loadings were constrained to be zero. In the ESEM model, all items were allowed to load on every factor and all factor loadings were estimated by imposing appropriate restrictions on the factor loading matrix and the factor covariance matrix (Asparouhov and Muthén, 2009; Marsh et al., 2010). An oblique geomin rotation was used in the ESEM model because the MSR factors are expected to covary and the geomin rotation criterion is the most effective criterion when the true factor loading structure is unknown (Asparouhov and Muthén, 2009).

To assess overall model fit, several criteria were used: the MLR chi-square statistic (Muthén and Muthén, 1998–2019), the comparative fit index (CFI; Bentler, 1990), the Tucker-Lewis index (TLI; Tucker and Lewis, 1973), the root mean square error of approximation (RMSEA; Steiger, 1990), and the standard root mean square residual (SRMR; Hu and Bentler, 1998). Values on the CFI and TLI that are > 0.90 and 0.95 are generally taken to reflect acceptable and excellent fits to the data (e.g., Marsh et al., 2010). For the RMSEA, values of 0.05 or less indicate a close fit, and 0.08 or less indicate an adequate fit (Browne and Cudeck, 1993). Values on the SRMR that are < 0.08 indicate an adequate fit (Hu and Bentler, 1998). Conventional multiple cut-off values (i.e., the CFI and TLI ≥ 0.90, the RMSEA ≤ 0.08, the SRMR ≤ 0.08) were considered minimum thresholds for accepting model fit. For the assessment of the fit of individual items, standardized factor loadings, residuals, and modification indices were carefully examined. The internal consistency of the MSRS responses was assessed using Cronbach's (Cronbach, 1951) coefficient alpha (α) and McDonald's (McDonald, 1999) coefficient omega (ω). For the second purpose, latent class factor analysis (LCFA) was conducted as taxometric analysis with *M*plus based on robust maximum likelihood estimation. Combining the strengths of both latent class analysis (LCA) and factor analysis, LCFA provides a factor analytical interval-scaled dimension with continuous scores on that factor (Muthén and Muthén, 1998–2019; Muthén, 2006). To aid in determining the number of classes, several criteria were used for assessment of model fit as a whole. These included the loglikelihood (logL), Akaike's information criterion (AIC; Akaike, 1987), Bayesian information criterion (BIC; Schwartz, 1978), and sample-size adjusted BIC (ABIC; Sclove, 1987). A model with both a high logL value and low AIC, BIC, and ABIC values is considered optimal (Muthén, 2006). Furthermore, a likelihood ratio test (LMR; Lo et al., 2001) was used to test k-1 versus k classes. A small *p*-value indicates that the model with k classes is favored over k-1 classes. A MANOVA was conducted separately for each sample to compare the CMP and MS-C scores across the groups identified in LCFA.

For the third purpose, correlation analysis, mediation analysis, and MANOVA were conducted for a part of the Singaporean sample to elaborate how individual's tendency for reinvestment is associated with other psychological variables. The MSRS dimensions (MS-C and CMP) are about individual's dispositional tendency for reinvestment in actions (i.e., movement-specific situations), whereas flow experience is considered to be the product of focused engagement in the activity. From a chronological perspective, therefore, mediation analysis was conducted on the data from a part of Singaporean sample (*n* = 53) participating in a competitive sport with the SPSS version of the PROCESS macro (Version 3.4.1; Hayes, 2018). The bootstrapping confidence interval (CI) approach was employed for inference about the direct, indirect, and total effects in the mediation models. To construct 95% CIs, bootstrapping was conducted with the percentile method as it is a good compromise test that is powerful but shows less Type I error inflation in smaller samples (Hayes and Scharkow, 2013). Fritz and MacKinnon (2007) suggested the sample size of 36 as a lower limit of the number of participants needed for a power of 0.80 when the mediation analysis is conducted with the percentile bootstrapping method and the effect sizes of indirect-related paths are large. Therefore, the mediation analysis was conducting in the present study by following the study by Kawabata and Chua (2021).

## Results

### Descriptive Analyses

The descriptive statistics of the MSRS item scores were presented in Table 1. For both samples, the item with the lowest score was Item 6 (MS-C). However, the items with the highest score were Item 5 (MS-C) and Item 9 (CMP) for Japanese and Singaporean samples, respectively.

**Table 1:** Descriptive statistics of item scores for Japanese and Singaporean samples.

			***M***	***SD***		***M***	***SD***
**Item**		**Japanese (*****n*** **= 236)**			**Singaporeans (*****n*** **= 328)**		
		**α**	**ω (95% CI)**		**α**	**ω (95% CI)**	
**Conscious motor processing**		0.75	0.78 (0.74–0.83)		0.75	0.49 (0.37–0.62)	
1	I remember the times when my movements have failed me.		4.06	1.50		4.21	1.33
3	I reflect about my movement a lot.		3.71	1.47		3.96	1.31
4	I try to think about my movements when I carry them out.		3.56	1.42		4.20	1.27
7	I am aware of the way my body works when I am carrying out a movement.		3.31	1.16		4.20	1.10
9	I try to figure out why my actions failed.		3.80	1.36		4.56	1.22
**Movement self-consciousness**		0.85	0.85 (0.82–0.89)		0.84^a^	0.72 (0.65–0.79)^a^	
2	If I see my reflection in a shop window, I will examine my movements.		3.92	1.47		3.95	1.38
5	I am self-conscious about the way I look when I am moving.		4.11	1.36		3.79	1.32
6	I sometimes have the feeling that I am watching myself move.		3.25	1.42		3.24	1.34
8	I am concerned about my style of moving.		3.94	1.35		3.81	1.32
10	I am concerned about what people think about me when I am moving.		3.87	1.41		3.44	1.42

a *The coefficients are based on 5 items. The coefficients for 4 items (Items 5, 6, 8, 10) are: α = 0.84, ω (95% CI) = 0.73 (0.66–0.70)*.

### CFA and ESEM

The overall fit of the 2-factor CFA model to the Japanese data was satisfactory according to all the overall fit indices (see Model 1 in Table 2). All standardized factor loadings were statistically significant, ranging from 0.45 (CMP Item 1) to 0.87 (MS-C Item 8). However, the latent correlation between the two factors was 0.86, indicated that the latent correlation was inflated due to the cross-loading of several items (Marsh et al., 2010). The corresponding 2-factor ESEM model also fit the Japanese data satisfactory (see Model 2). All standardized factor loadings on the targeted factor were statistically significant, ranging from 0.22 (CMP Item 1) to 0.86 (MS-C Item 5). The sizes of non-targeted cross-loadings were smaller for all items, except for CMP Item 1. The latent correlation in this model was 0.57 and much lower than the latent correlation in the corresponding CFA model (Model 1). These results clearly showed that the latent correlation in the CFA model was inflated due to the cross-loading of several items. As shown in Table 1, alpha and omega coefficients were above 0.75, indicated that both CMP and MS-C scores of the Japanese sample were internally consistent.

**Table 2:** Summary of goodness-of-fit statistics for specified models.

	**Model Description**	**MLRχ^2^**	***df***	**CFI**	**TLI**	**SRMR**	**RMSEA**	**(90% CI)**
**Japanese sample (*****n*** **= 234)**								
M1	2-factor CFA (10 items)	90.20	34	0.938	0.918	0.054	0.084	0.063–0.105
M2	2-factor ESEM (10 items)	73.49	26	.957	0.926	0.043	0.088	0.065–0.112
**Singaporean sample (*****n*** **= 328**)								
M1	2-factor CFA (10 items)	125.90	34	0.908	0.878	0.060	0.091	0.074–0.108
M2	2-factor ESEM (10 items)	104.53	26	0.936	0.890	0.039	0.096	0.077–0.116
M3	2-factor CFA (M1—Item 2)	57.58	26	0.962	0.948	0.046	0.061	0.040–0.082
M4	2-factor ESEM (M2—Item 2)	42.32	19	0.977	0.957	0.028	0.061	0.036–0.086

*CFA, confirmatory factor analysis; ESEM, exploratory structural equation modeling; MLRχ^2^, Mplus robust maximum likelihood estimation; CFI, robust comparative fit index; TLI, tucker-lewis index; SRMR, standard root mean square residual; RMSEA, robust root mean square error of approximation*.

For the Singaporean sample, neither the 2-factor CFA model nor the 2-factor ESEM model fit the data satisfactorily (Models 1 and 3 in Table 2). Inspection of the item factor loadings in Model 2 revealed that Item 2 loaded more on its non-target factor (CMP) than its target factor (MS-C). Therefore, Item 2 was removed and CFA and ESEM were conducted with nine items. Both 2-factor CFA and ESEM models (Models 3 and 4) fit the data very well. In Model 3, all standardized factor loadings were statistically significant, ranging from 0.52 (CMP Item 1) to 0.83 (CMP Item 4), and the latent correlation between the two factors was 0.61. In Model 4, all standardized factor loadings on the targeted factor were statistically significant, ranging from 0.58 (CMP Item 1) to 0.79 (MS-C Item 5). The sizes of non-targeted cross-loadings were smaller for all items, and the latent correlation in this model was 0.33. Alpha and omega coefficients were above 0.72, except for the omega coefficient for CMP (0.49). However, the corresponding alpha coefficient was 0.75 (see Table 1). Thus, both CMP and MS-C scores of the Singaporean sample were also considered internally consistent.

### LCFA

A series of LCFAs were conducted to find homogeneous groups of individuals and obtain a factor mean for each class. The fit statistics for LCFA models are provided in Table 3. For the Japanese sample, the logL values continued to increase as the number of classes rose and leveled off when going from four to five classes. For the three information indices, the values for AIC, BIC, and ABIC were consistently at their optimum at four classes. Furthermore, the LMR test was significant for the four-class solution, pointing to four classes. According to these results, it was concluded that the four-class solution was preferred for the Japanese sample. Following the same assessment criteria, three classes were considered optimum for the Singaporean sample.

**Table 3:** Goodness-of-fit statistics for LCFA models on different numbers of classes.

**No.Classes**	**logL**	**No.Parameter**	**AIC**	**BIC**	**ABIC**	***p*LMR**
**Japanese sample (*****n*** **= 234)**						
2	−629.662	7	1273	1298	1275	0.0000
3	−598.113	9	1214	1245	1217	0.0000
4	−588.272	11	1198	1237	1202	0.0000
5	−585.965	13	1198	1243	1202	0.4983
**Singaporean sample (*****n*** **= 328)**						
2	−878.716	7	1771	1798	1776	0.0000
3	−870.794	9	1760	1794	1765	0.0541
4	−867.495	11	1757	1799	1764	0.1779
5	−864.152	13	1754	1804	1762	0.1157

*logL, loglikelihood; AIC, akaile's information criterion; BIC, bayesian information criterion; ABIC, adjusted Bayesian information criterion; pLMR, p values for the Lo-Mendell-Rubin likelihood ratio test for k vs. k-1 classes*.

**Figure 1:**
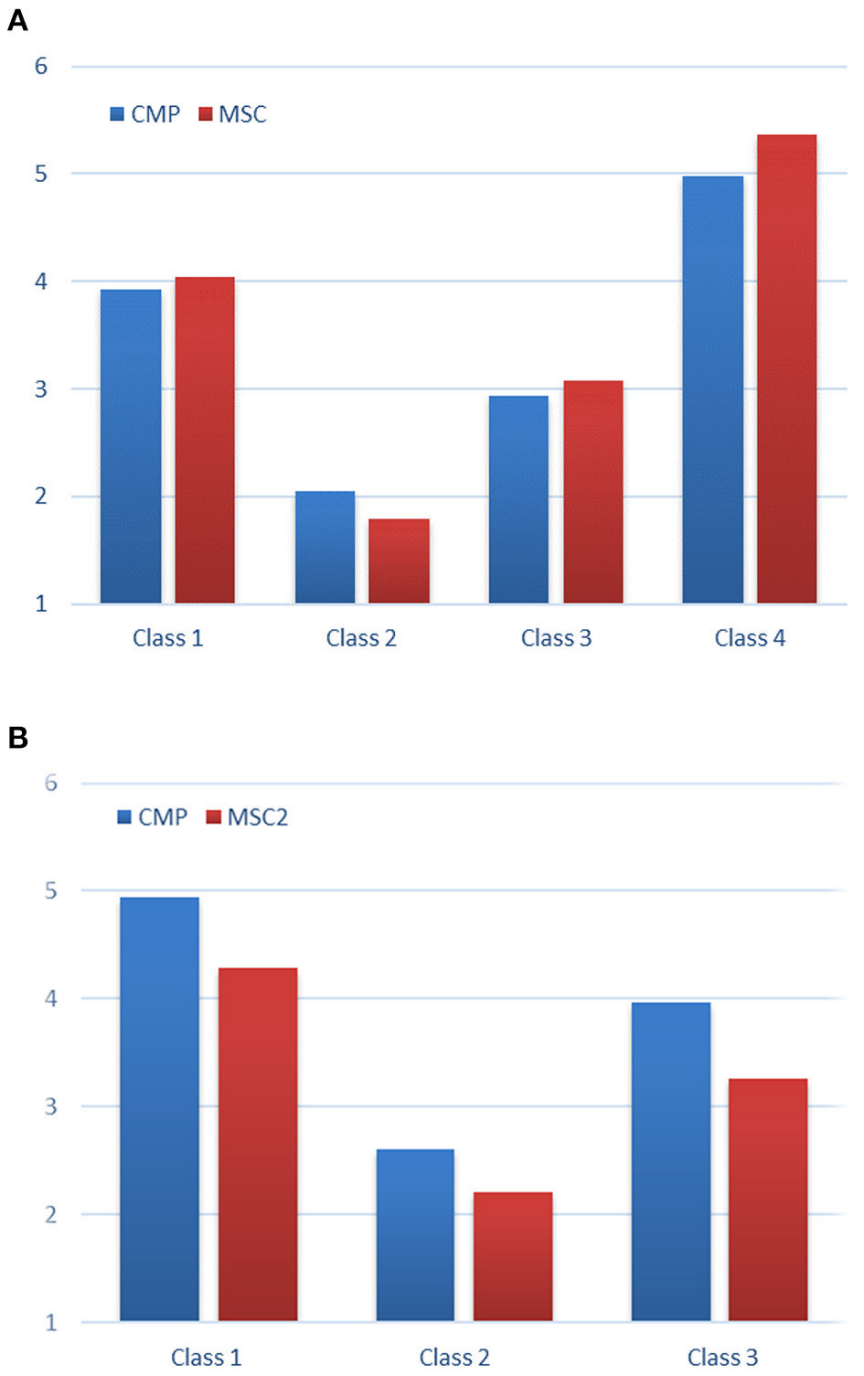
Latent class factor analysis profile. **(A)** The Japanese sample (*n* = 234). The estimated class percentages are Class 1 (*n* = 131, 56.0%), Class 2 (*n* = 21, 9.0%), Class 3 (*n* = 48, 20.5%), Class 4 (*n* = 34, 14.5%). **(B)** The Singaporean sample (*n* = 328). The estimated class percentages are Class 1 (*n* = 127, 38.7%), Class 2 (*n* = 28, 8.5%), Class 3 (*n* = 173, 52.7%). CMP, conscious motor processing; MS-C, movement self-consciousness. The *x*-axis lists the identified four classes and the y-axis shows the item-average scores of CMP and MS-C.

Figures 1A,B show the LCFA profiles for the Japanese and Singaporean samples. The x-axis lists the identified four classes, while the y-axis shows the item-average scores of CMP and MS-C. For both samples, the patterns of the item-average scores for CMP and MS-C were found parallel among the classes. It means that the profiles of the two scores were similar qualitatively (profile shape) but different quantitatively (profile level) (see Marsh et al., 2009). MANOVAs on the CMP and MS-C scores indicated that the main effect of the classes was significant for both samples, (Japanese: *F*_[3, 230]_ = 144.34, *p* < 0.001, $\eta_{p}^{2}$ = 0.65; Singaporeans: *F*_[2, 325]_ = 197.18, *p* < 0.001, $\eta_{p}^{2}$ = 0.55). Separate univariate ANOVAs revealed that CMP and MS-C scores were significantly different across the classes for both samples. Classes 4 and 2 were the highest and lowest investment groups for the Japanese sample (Figure 1A), whereas Classes 3 and 2 were the highest and lowest investment groups for the Singaporean sample (Figure 1B).

### Associations Between the MSRS Dimensions and Relevant Psychological Traits

Correlation analysis, mediation analysis, and MANOVA were conducted for a part of Singaporean sample (*n* = 53) who participated in a competitive sport. Table 4 presents a summary of correlations between the MSRS subscale scores and relevant psychological scale scores. CMP was significantly positively associated with mental toughness, vitality, intrinsic regulation, integrated regulation, mastery-approach and task goal orientations, and dispositional flow. On the other hand, MS-C was significantly positively related with vitality, stress, mastery-avoidance goal orientation. For the participants who competed at the national or international revel (*n* = 18), CMP was significantly positively associated with intrinsic regulation and mastery-approach orientation, whereas MS-C was significantly positively related with external regulation.

**Table 4:** Pearson's correlations between the MSRS and relevant psychological scores for a part of the Singapore sample (*n* = 53).

**Subscale**	**Mental toughness**	**Vitality**	**Stress**	**Intrinsic regulation**	**Integrated regulation**	**External regulation**	**Mastery Approach**	**Masteryavoidance**	**Ego**	**Task**	**Flow**
**All (*****n*** **= 53)**											
CMP	**0.34**	**0.34**	0.03	**0.34**	**0.27**	0.20	**0.44**	0.20	−0.13	**0.38**	**0.30**
MS-C	0.18	**0.36**	**0.29**	0.09	0.19	0.20	0.24	**0.21**	0.22	0.09	−0.07
**National or International level (*****n*** **= 18)**											
CMP	0.03	0.29	0.31	**0.48**	0.33	0.30	**0.62**	0.24	−0.18	0.31	0.27
MS-C	−0.31	0.04	0.39	0.36	0.20	**0.52**	0.22	−0.12	0.04	−0.01	−0.15
**School or recreational level (*****n*** **= 35)**											
CMP	**0.55**	**0.38**	−0.21	0.25	0.25	0.04	**0.36**	0.23	−0.14	**0.41**	0.31
MS-C	**0.40**	**0.52**	0.25	−0.01	0.20	−0.02	0.25	0.32	0.30	0.14	−0.02

*MSRS, the movement-specific reinvestment scale; CMP, conscious motor processing; MS-C, movement self-consciousness. All correlations bolded were significant at p ≤ 0.05*.

**Figure 2:**
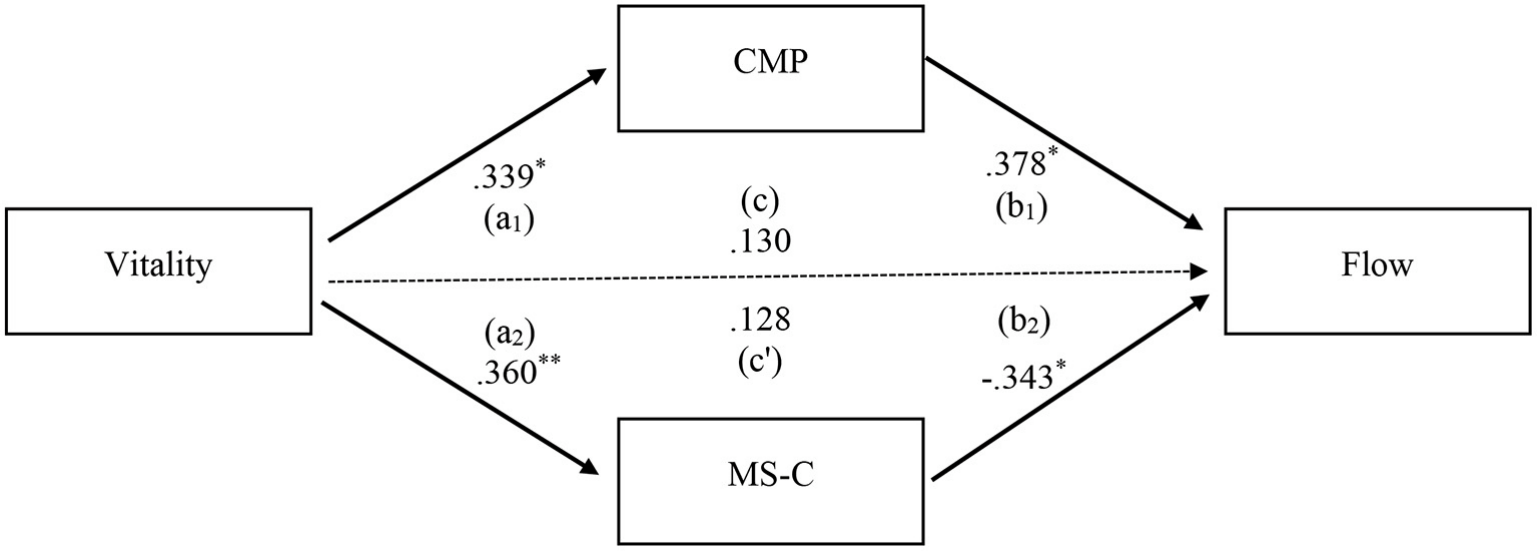
Multiple mediation model for the association between vitality and flow. CMP, conscious motor processing; MSC, movement self-consciousness. Standardized coefficients (a_1_, a_2_, b_1_, b_2_) from a bootstrap procedure are shown along the paths. The total effect of the antecedent variable (c) and the direct effect of the antecedent variable on the outcome variable (c'). Solid paths are significant (*p* < 0.05) and dotted paths are non-significant (*p* > 0.05). **p < 0.01, *p < 0.05.

Based on the results of correlations reported above, only vitality was significantly associated with both MSRS dimensions. Therefore, a mediation analysis was conducted for the association between vitality and flow by using the parallel multiple mediator model (Figure 2), in which multiple mediators are not directly related to each other. All standardized coefficients related to CMS and MS-C (a1, a2, b1, b2) were statistically significant. However, the indirect effect of MS-C (a2b2 = −0.123) was only statistically significant as the CI did not include zero (see Table 5). Although the absolute sizes of the indirect effects of CMP and MS-C were similar (i.e., |0.128| and |0.123|), the indirect effect of CMP was not statistically significant as the CI included zero (−0.009, 0.296). In the parallel multiple mediator model, a specific indirect effect is interpreted as the estimated amount of the effect that the independent variable has on the dependent variable through the mediator by controlling for all other mediators in the model (Hayes, 2018). Thus, the significant indirect effect of −0.123 (CI: −0.298, −0.005) means that the association between subjective vitality and the frequency of flow experience in sport was negatively affected by the higher MS-C propensity.

**Table 5:** Indirect effects of subjective vitality on dispositional flow through movement-specific reinvestment dimensions (*n* = 53).

**IV**	**Parallel mediators**	**DV**	**IDE_**s**_**	***SE_***b***_***	**95% CI*_***b***_* Lower, Upper**
Vitality	CMP	Flow	0.128	0.079	−0.009, 0.296
	MS-C		−0.123	0.076	−0.298, −0.005^*^

*IV, independent variable; Vitality,subjective vitality; CMP, conscious motor professing; MS-C, movement self-consciousness; Flow, dispositional flow; DV, dependent variable; IDE_s_, standardized indirect effect; SE_b_, bootstrapped standard error; CI_b_, bootstrapped 95% confidence interval with the percentile method.*

* *Confident interval excludes zero*.

According to the three classes identified by LCFA, Class 2 in the Singaporean sub-sample included one participant only and therefore Class 2 was combined into Class 3. Consequently, a MANOVA was conducted on the relevant psychological scores across two groups (Class 1: *n* = 15; Class 3: *n* = 38). It was found that Class 1 had significantly higher vitality and mental toughness scores (vitality: *M* = 4.99, *SD* = 0.80, *F*_[1, 51]_ = 4.42, *p* = 0.04, $\eta_{p}^{2}$ = 0.08); mental toughness: *M* = 4.04, *SD* = 0.60, *F*_[1, 51]_ = 3.95, *p* = 0.05, $\eta_{p}^{2}$ = 0.08, $\eta_{p}^{2}$ = 0.07) than Class 3 (vitality: *M* = 4.33, *SD* = 1.05; mental toughness: *M* = 3.76, *SD* = 0.41).

## Discussion

The present study aimed to examine (a) if the movement-specific reinvestment responses should be represented as two dimensional constructs, (b) whether dichotomization of the movement-specific reinvestment responses are appropriate, and (c) how the dimensions of movement-specific reinvestment are associated differently with relevant psychological concepts.

In the first analysis, the factor structure of the MSRS responses was examined by conducting CFA and ESEM with cross-language samples. Comparisons of the latent correlation in the 2-factor CFA and ESEM models clearly showed that for both Japanese and Singaporean samples, the latent correlation in the CFA model was inflated due to item cross-loadings. However, the latent correlation in the ESEM model indicated that CMP and MS-C were empirically distinguishable and positively associated at the moderate level for the Japanese and Singaporean samples. This result is important for how to use the MSRS scores. Although CMP and MS-C were positively associated, the strength of the relationship was at the moderate level. Thus, the scores of the two dimensions should be kept separately rather than combining into a single score.

Through a series of LCFA, four and three classes were identified for the Japanese and Singaporean samples, respectively. It was found that CMP and MS-C scores were significantly different across the classes for both samples. These results supported the validity of the classes identified through LCFA. A conclusion to be drawn from LCFA is that individual's tendency for reinvestment measured by the MSRS falls into a few categories, which were more than two. The number of latent classes identified in the Japanese sample was four, whereas three classes were identified in the Singaporean sample. This difference might be caused by employing different languages (i.e., English and Japanese versions of the MSRS), individual differences, or type of physical activities (e.g., Kawabata and Mallett, 2012, Kawabata and Evans, 2016). However, the numbers of latent classes identified were similar across the two language/cultural samples. Given that this question has not been examined in the previous studies on the MSRS responses by conducting taxometric analyses such as LCFA, the findings from the present study represents a unique and significant contribution of the current study to the literature. For both samples, the patterns of the item-average scores for the two movement-specific reinvestment dimensions were parallel among the classes. Based on the results of LCFA across the two language/cultural samples (Figures 1A,B), the item-average scores of 2, 3, 4, 5 are proposed for the two dimensions to categorize the MSRS responses into three to four groups (e.g., low, middle, and high investors). For example, if your sample size is not large and want to compare the MSRS responses between two groups, you may consider using the item-average score of 3 for the two dimensions to have two groups (low and high investors). Instead, if you collect data from a large sample with the English version of the MSRS, you may consider employing the item-average scores of 2, 3, 4 to have three groups (low. middle, high investors).

As for the relationships between the MSRS dimensions and relevant psychological traits in the Singaporean athlete sample (*n* = 53), CMP was positively associated with mental toughness, vitality, intrinsic regulation, integrated regulation, mastery-approach and task goal orientations, and dispositional flow. On the other hand, MSC was positively related with vitality, stress, mastery-avoidance goal orientation.

CMP is consistent with reinvestment of task-relevant declarative knowledge (Masters and Maxwell, 2008). In the learning process from a declarative knowledge stage, performers pay much attention to a procedural knowledge and control their movements in order to accomplish the task. From the perspectives of goal orientations and motivational style, this learning process is related to mastering the task by focusing the task itself. Thus, it is reasonable to see CMP was positively associated with self-determined motivations (intrinsic and integrated regulations), mastery-approach, task goal orientations. Given that flow is a state in which individuals are engaging in a task at hand with focused and intensive attention, a positive relation between CMP and dispositional flow is also considered sensible. Mental toughness is a personality trait that determines how individuals deal with challenges, stressors and pressures effectively (Clough and Strycharczyk, 2015). Given that the correlation between CMP and mental toughness was not significant for participants (*n* = 18) who competed their sport at national and international levels, the significant positive correlation observed for the entire athlete sample (*n* = 53) was because of the participants playing sports at school or recreational level (*n* = 35). MS-C is individual's tendency for concerning about style of movement and about what other people think about one's movement. Given that mastery-avoidance goal orientation is a focus on avoiding task-based or intrapersonal incompetence (Elliot and Murayama, 2008), individuals with higher the MS-C score would show higher mastery-avoidance goal orientation. Thus, it is reasonable to see MS-C was positively related with stress and mastery-avoidance goal orientation.

According to the mediation analysis, it was found that the association between subjective vitality and the frequency of flow experience in sport was negatively affected by the higher MS-C propensity. This was due to the negative path from MS-C to dispositional flow. In a flow state, individuals are doing a task with focused and intensive involvement and are not concerned how they are perceived by others. Therefore, the negative path from MS-C to dispositional flow is considered sensible. Furthermore, the parallel multiple mediator model indicated that CMP and MS-C, respectively were positively and negatively related to dispositional flow although both dimensions were positively associated with subjective vitality. Given that all the numerical variables were centered in the parallel multiple mediator model, multicollinearity is unlikely to cause the opposite directions of the correlations between the two MSRS dimensions and dispositional flow. This is another important evidence for keeping the scores of two MSRS dimensions separately to understand the individual's tendency for reinvestment as they are differently associated with important psychological constructs such as flow.

These correlation results indicated that at the trait level, CMP and MS-C were associated with positive and negative psychological constructs, respectively. However, caution is warranted to interpret the correlation results. First, the sample size employed for the correlation and mediation analyses are not large and the results cannot be generalized. More importantly, reinvestment researchers argued that involvement of conscious monitoring and control mechanisms in motor processes depends on situational contexts, such as psychological pressure or individual personality differences (Masters and Maxwell, 2008; Malhotra et al., 2015). The present study focused on individual personality differences by using trait-level measures (except for the DASS-21). Thus, situational contexts (e.g., psychological pressure) should be included in a study to understand the concept of reinvestment well.

In the present study, the validity of the classes identified through LCFA was examined by conducting MANOVA. Nylund-Gibson et al. (2019) recommended using new methods such as BCH (Bolck-Croon-Hagenaars) method (Bolck et al., 2004) in *M*plus to examine the relationship between latent class membership and distal outcomes. However, the BCH method was not employed in the present study as the association between investment groups and other psychological scores was examined based on the data from the Singaporean sub-sample (*n* = 53). The recommended method should be used in the future study.

## Conclusions

The findings of the present study supported the two-dimensional representation of the movement-specific reinvestment responses; however, did not fully support the practice of dichotomization of the MSRS responses. It was also indicated that at the trait level, CMP and MSC were associated with positive and negative psychological constructs, respectively. Although the two MSRS dimensions are moderately and positively related, their scores should be analyzed separately to properly understand the individual's tendency for reinvestment. The present study could serve as a catalyst for future studies to understand the complex mechanism of reinvestment.

## Data Availability Statement

The datasets used and analyzed during the current study are available from the corresponding author on reasonable request.

## Ethics Statement

Ethics approval for the study was obtained from the Institutional Review Board at Nanyang Technological University, Singapore (Ref No: IRB-2013-11-004), and the guidelines of Nangyang Technological University for Ethical reviews of research involving humans were followed in the conduct of the study.

## Author Contributions

MK developed the study concept, conducted all the data analyses, and drafted the manuscript. Data were collected by MK or KI. The publication of the article was financially supported by a research grant KI had received. All authors approved the final version of the manuscript for submission.

## Conflict of Interest

The authors declare that the research was conducted in the absence of any commercial or financial relationships that could be construed as a potential conflict of interest.

## Publisher's Note

All claims expressed in this article are solely those of the authors and do not necessarily represent those of their affiliated organizations, or those of the publisher, the editors and the reviewers. Any product that may be evaluated in this article, or claim that may be made by its manufacturer, is not guaranteed or endorsed by the publisher.

## References

[B1] Akaike H. (1987). Factor analysis and AIC. Psychometrika 52, 317–332. 10.1007/BF02294359

[B2] Asparouhov T. Muthén B. (2009). Exploratory structural equation modeling. Struct. Equ. Model. 16, 397–438. 10.1080/10705510903008204

[B3] Beilock S. L. Carr T. H. (2001). On the fragility of skilled performance: what governs choking under pressure? J. Exp. Psychol. Gen. 130, 701–725. 10.1037/0096-3445.130.4.701 11757876

[B4] Bentler P. M. (1990). Comparative fit indexes in structural models. Psychol. Bull. 107, 238–246. 10.1037/0033-2909.107.2.238 2320703

[B5] Bolck A. Croon M. Hagenaars J. (2004). Estimating latent structure models with categorical variables: one-step versus three-step estimators. Polit. Anal. 12, 3–27. 10.1093/pan/mph001

[B6] Browne M. W. Cudeck R. (1993). Alternative ways of assessing model fit, in Testing Structural Equation Models, eds Bollen K. A. Long J. S. (Newbury Park, CA: Sage), 445–455.

[B7] Clough P. Strycharczyk D. (2015). Developing Mental Toughness: Coaching Strategies to Improve Performance, Resilience and Wellbeing. London, UK: Kogan Page.

[B8] Cronbach L. J. (1951). Coefficient alpha and the internal structure of tests. Psychometrika 16, 297–334. 10.1007/BF02310555

[B9] Duda J. L. Hayashi C. T. (1998). Measurement issues in cross-cultural research within sport and exercise psychology, in Advances in Sport and Exercise Psychology Measurement, ed Duda J. L. (Morgantown, WV: Fitness Information Technology), 471–483.

[B10] Duda J. L. Nicholls J. G. (1992). Dimensions of achievement motivation in schoolwork and sport. J. Educ. Psychol. 84, 290–299. 10.1037/0022-0663.84.3.290

[B11] Duda J. L. Papaioannou A. G. Appleton P. R. Quested E. Krommidas C. (2014). Creating adaptive motivational climates in sport and physical education, in Routledge Companion to Sport and Exercise Psychology: Global Perspectives and Fundamental Concepts, eds Papaioannou A. G. Hackfort D. (New York, NY: Routledge), 544–558.

[B12] Elliot A. J. Murayama K. (2008). On the measurement of achievement goals: critique, illustration, and application. J. Educ. Psychol. 100, 613–628. 10.1037/0022-0663.100.3.613

[B13] Fritz M. S. MacKinnon D. P. (2007). Required sample size to detect the mediated effect. Psychol. Sci. 18, 233–239. 10.1111/j.1467-9280.2007.01882.x 17444920 PMC2843527

[B14] Hayes A. F. (2018). Introduction to Mediation, Moderation, and Conditional Process Analysis: A Regression-Based Approach, 2nd Edn. New York, NY: Guilford Press.

[B15] Hayes A. F. Scharkow M. (2013). The relative trustworthiness of inferential tests of the indirect effect in statistical mediation analysis: does method really matter? Psychol. Sci. 24, 1918–1927. 10.1177/0956797613480187 23955356

[B16] Hu L. Bentler P. M. (1998). Fit indices in covariance structure modeling: sensitivity to underparameterized model misspecification. Psychol. Methods 3, 424–453. 10.1037/1082-989X.3.4.424

[B17] Jackson S. A. Martin A. J. Eklund R. C. (2008). Long and short measures of flow: the construct validity of the FSS-2, DFS-2, and new brief counterparts. J. Sport Exerc. Psychol. 30, 561–587. 10.1123/jsep.30.5.561 18971512

[B18] Kawabata M. Chua K. L. (2021). A multiple mediation analysis of the association between asynchronous use of music and running performance. J. Sports Sci. 39, 131–137. 10.1080/02640414.2020.1809153 32809899

[B19] Kawabata M. Evans R. (2016). How to classify who experienced flow from who did not based on the Flow State Scale-2 scores: a pilot study of latent class factor analysis. Sport Psychol. 30, 267–275. 10.1123/tsp.2014-0053

[B20] Kawabata M. Mallett C. J. (2011). Flow experience in physical activity: examination of the internal structure of flow from a process-related perspective. Motiv. Emotion 35, 393–402. 10.1007/s11031-011-9221-1

[B21] Kawabata M. Mallett C. J. (2012). Interpreting the dispositional Flow Scale-2 scores: a pilot study of latent class factor analysis. J. Sports Sci. 30, 1183–1188. 10.1080/02640414.2012.695083 22709370

[B22] Kawabata M. Mallett C. J. Jackson S. A. (2008). The Flow State Scale-2 and dispositional Flow Scale-2: examination of factorial validity and reliability for Japanese adults. Psychol. Sport Exerc. 9, 465–485. 10.1016/j.psychsport.2007.05.005

[B23] Kawabata M. Pavey T. G. Coulter T. J. (2020). Evolving the validity of a mental toughness measure: refined versions of the Mental Toughness Questionnaire-48. Stress Health 37, 378–391. 10.1002/smi.3004 33145967

[B24] Kawabata M. Yamazaki F. Guo D. W. Chatzisarantis N. L. D. (2017). Advancement of the subjective vitality scale: examining alternative measurement models for Japanese and Singaporeans. Scand. J. Med. Sci. Sports 27, 1793–1800. 10.1111/sms.12760 27704634

[B25] Laborde S. Dosseville F. Kinrade N. (2014). Decision-specific reinvestment scale: an exploration of its construct validity, and association with stress and coping appraisals. Psychol. Sport Exerc. 15, 238–245. 10.1016/j.psychsport.2014.01.004

[B26] Laborde S. Musculus L. Kalicinski M. Klämpfl M. K. Kinrade N. Lobinger B. H. (2015). Reinvestment: examining convergent, discriminant, and criterion validity using psychometric and behavioral measures. Psychol. Sport Exerc. 78, 77–87. 10.1016/j.paid.2015.01.020

[B27] Ling F. C. M. Maxwell J. Masters R. S. W. McManus A. M. Polman R. C. J. (2016). Psychometric properties of the movement-specific reinvestment scale for Chinese children. Int. J. Sport Exerc. Psychol. 14, 227–239. 10.1080/1612197X.2015.1016087

[B28] Lo Y. Mendell N. Rubin D. (2001). Testing the number of components in a normal mixture. Biometrika 88, 767–778. 10.1093/biomet/88.3.767

[B29] Lovibond S. H. Lovibond P. F. (1995). Manual for the Depression Anxiety & Stress Scales, 2nd Edn. Sydney, NSW: Psychology Foundation. 10.1037/t39835-000

[B30] MacCallum R. C. Zhang S. Preacher K. J. Rucker D. D. (2002). On the practice of dichotomization of quantitative variables. Psychol. Methods 7, 19–40. 10.1037/1082-989X.7.1.19 11928888

[B31] Malhotra N. Poolton J. M. Wilson M. R. Omuro S. Masters R. S. W. (2015). Dimensions of movement specific reinvestment in practice of a golf putting task. Psychol. Sport Exerc. 18, 1–8. 10.1016/j.psychsport.2014.11.008

[B32] Mallett C. J. Kawabata M. Newcombe P. (2007). Progressing measurement in sport motivation with the SMS-6: a response to Pelletier, Vallerand, and Sarrazin. Psychol. Sport Exerc. 8, 622–631. 10.1016/j.psychsport.2007.05.001

[B33] Marsh H. W. Lüdtke O. Muthén B. Asparouhov T. Morin A. J. S. Trautwein U.. (2010). A new look at the big-five factor structure through exploratory structural equation modeling. Psychol. Assess. 22, 471–491. 10.1037/a0019227 20822261

[B34] Marsh H. W. Lüdtke O. Trautwein U. Morin A. J. S. (2009). Classical latent profile analysis of academic self-concept dimensions: synergy of person and variable-centered approaches to theoretical models of self-concept. Struct. Equ. Model. 16, 191–225. 10.1080/10705510902751010

[B35] Masters R. S. W. (1992). Knowledge, knerves and know-how: the role of explicit versus implicit knowledge in the breakdown of a complex motor skill under pressure. Br. J. Psychol. 83, 343–348. 10.1111/j.2044-8295.1992.tb02446.x

[B36] Masters R. S. W. Eves F. F. Maxwell J. P. (2005). Development of a movement specific reinvestment scale, in Proceeding of the ISSP 11th World Congress of Sport Psychology, eds Morris T. Gordon S. Hanrahan S. Ievleva L. Kolt G. Tremayne P. (Sydney, NSW: ISSP).

[B37] Masters R. S. W. Maxwell J. P. (2008). The theory of reinvestment. Int. Review Sport Exerc. Psychol. 1,160–183. 10.1080/17509840802287218

[B38] Masters R. S. W. Polman R. C. J. Hammond N. V. (1993). 'Reinvestment': a dimension of personality implicated in skill breakdown under pressure. Pers. Individ. Dif. 14, 655–666. 10.1016/0191-8869(93)90113-H

[B39] McDonald R. P. (1999). Test Theory: A Unified Treatment. Mahwah, NJ: Erlbaum.

[B40] Muthén B. (2006). Should substance use disorders be considered as categorical or dimensional? Addiction 101(1 Suppl. 1), 6–16. 10.1111/j.1360-0443.2006.01583.x 16930156

[B41] Muthén L. K. Muthén B. (1998–2019). Mplus User's Guide, 8th Edn. Los Angeles, CA: Muthén & Muthén.

[B42] Ntoumanis N. Mallett C. J. (2014). Motivation in sport, in Routledge Companion to Sport and Exercise Psychology: Global Perspectives and Fundamental Concepts, eds Papaioannou A. G., Hackfort D., (New York: Routledge), 67–82.

[B43] Nylund-Gibson K. Grimm R. P. Masyn K. E. (2019). Prediction from latent classes: a demonstration of different approaches to include distal outcomes in mixture models. Struct. Equ. Model. 26, 967–985. 10.1080/10705511.2019.1590146

[B44] Pelletier L. G. Rocchi M. A. Vallerand R. J. Deci E. L. Ryan R. M. (2013). Validation of the revised sport motivation scale (SMS-II). Psychol. Sport Exerc. 14, 329–341. 10.1016/j.psychsport.2012.12.002

[B45] Roger D. Masters R. S. W. (1997). The development and evaluation of an emotion control training programme for sex offenders. Leg. Criminol. Psychol. 2, 51–64. 10.1111/j.2044-8333.1997.tb00332.x

[B46] Ryan R. M. Frederick C. M. (1997). On energy, personality and health: subjective vitality as a dynamic reflection of well-being. J. Pers. 65, 529–565. 10.1111/j.1467-6494.1997.tb00326.x 9327588

[B47] Schwartz G. (1978). Estimating the dimension of a model. Ann. Stat. 6, 461–464. 10.1214/aos/1176344136

[B48] Sclove L. (1987). Application of model-selection criteria to some problems in multivariate analysis. Psychometrika 52, 333–343. 10.1007/BF02294360

[B49] Steiger J. H. (1990). Structural model evaluation and modification: an interval estimation approach. Multivariate Behav. Res. 25, 173–180. 10.1207/s15327906mbr2502_4 26794479

[B50] Tucker L. R. Lewis C. (1973). A reliability coefficient for maximum likelihood factor analysis. Psychometrika 38, 1–10. 10.1007/BF02291170

[B51] Wulf G. Töllner T. Shea C. H. (2007). Attentional focus effects as a function of task difficulty. Res. Q. Exerc. Sport 78, 257–264. 10.1080/02701367.2007.10599423 17679499

[B52] Clough P. Earle K. Sewell D. (2002). Mental toughness: the concept and its measurement. In Cockerill I. (Ed.), Solutions in Sport Psychology. London: Thomson, 32–43.

[B53] Bentler P. M. (2006). EQS 6 structural equations program manual. Encino, CA: Multivariate Software.

